# A Methodological Review of Meditation Research

**DOI:** 10.3389/fpsyt.2014.00074

**Published:** 2014-07-01

**Authors:** John W. Thomas, Marc Cohen

**Affiliations:** ^1^School of Health Sciences, RMIT University, Bundoora, VIC, Australia

**Keywords:** meditation, consciousness, meditation states, methodology, subjective measures

## Abstract

Despite over 50 years of research into the states of consciousness induced by various meditation practices, no clear neurophysiological signatures of these states have been found. Much of this failure can be attributed to the narrow range of variables examined in most meditation studies, with the focus being restricted to a search for correlations between neurophysiological measures and particular practices, without documenting the content and context of these practices. We contend that more meaningful results can be obtained by expanding the methodological paradigm to include multiple domains including: the cultural setting (“the place”), the life situation of the meditator (“the person”), details of the particular meditation practice (‘the practice’), and the state of consciousness of the meditator (“the phenomenology”). Inclusion of variables from all these domains will improve the ability to predict the psychophysiological variables (“the psychophysiology”) associated with specific meditation states and thus explore the mysteries of human consciousness.

## Introduction

The term “meditation” refers to mental and emotional control practices from a number of cultural contexts including those of Christianity and Islam, yet is most frequently applied to those originating in the Eastern spiritual traditions of India, Tibet, China, and Japan. Meditation has been adopted in western countries both as a spiritual practice and a mind–body therapeutic intervention (1). The effects of meditation can be divided into – the lasting (therapeutic) changes in the person (traits) – and the immediate experiences during the meditation practice (states) (2). This review will focus on meditation states.

The states of consciousness induced by meditation practices have been extensively investigated from both a practical and a theoretical perspective. A practical question is: do meditation states have specific psycho-physiological characteristics that distinguish them from other mind–body therapeutic interventions, e.g., relaxation? (3, 4). As meditation practices were originally devised as a path to “higher consciousness,” a more theoretical question is: do meditation practices induce specific states of (altered) consciousness, distinguishable from other states of consciousness such as sleep (5) or hypnosis? (6).

### The practice of meditation

Given the wide range of practices called “meditation,” issues of definition continue to hamper research in this area, as predicted in an early review of the area (4). A recent review of Lutz et al. (7) suggests that the limited contributions made to date by neuroscientific studies of meditation can be attributed, in part, to differences in the studied meditative states (7).

In an extensive review of meditation practices in healthcare, the US Department of Health and Human Services found a definitive taxonomy of meditation practices was not possible due to the lack of specificity of the concept of meditation (8). An attempt to address this issue led to a generalized definition of meditation considered suitable for research purposes arising from the consensus of a panel of experts using Delphi method. Essential components of this definition were: (a) a defined technique, (b) logic relaxation, and (c) a self-induced state/mode. Non-essential but important components were: (d) involve a state of psychophysical relaxation somewhere in the process, (d) use a self-focus skill or anchor, (e) involve an altered state/mode of consciousness, (f) be imbedded in a religious/spiritual/philosophical context or (g) involve an experience of mental silence (9). While encompassing most variations of meditation practices, this definition does not provide specific dimensions on which particular practices can be classified.

Rather than exploring correlations between EEG measures and a vaguely defined “meditation state,” Ulrich Ott advocated that testable hypotheses be formulated, specifying the relationship of various cognitive processes to frequency and topographic EEG maps. This research agenda requires that meditation practices be more closely examined and that traditional instructions be expressed in the language of cognitive psychology (10). The most frequently used distinction for this purpose has been the focus of attention (4). Using this dimension, and based on traditional meditation texts and modern neuroscientific conceptions within the Buddhist tradition, Lutz et al. (11) proposed two broad categories for meditation practices: “focused attention” (FA) and “open monitoring” (OM), from which operational definitions could be derived. The cognitive tasks associated with each category can then be related to neurophysiological activity and brain location and testable hypotheses derived.

### The psychophysiology of meditation

Although meditation is inherently a subjective practice (4), early calls for research studies to include phenomenological data (12) have largely gone unheeded. Fell et al. (13) attempted to answer the question: “are meditation-related brain/mind states unique?” based on an examination of existing evidence. They concluded that early stages of meditation may overlap with other states of consciousness, e.g., relaxation, but at advanced levels of practice, the states attained are unique. They note, however, there are, as yet, few empirical studies of advanced meditators and so any conclusions remain tentative.

Walsh (14) saw phenomenological changes in meditation states as the very *raison d’etre* for meditation and suggested two approaches – rating scales for groups of subjects and intensive single-case studies. However, few studies to date have included phenomenological data. The predominant research design has been within-subject comparisons of psycho-physiological measures from participants engaged in a particular meditation practice to measures taken in a baseline state.

Such studies have led to brain activity during meditation practice being extensively explored, with the EEG being the most commonly used technology before the advent of neuroimagery. In line with the western emphasis on “objective” measures, most reports devote the great majority of their content to the technical aspects of psycho-physiological data collection and analysis. Yet, after 50 years of this approach to scientific research into meditation, there is still no consensus about the neurophysiological processes underlying meditation practice (2).

### The phenomenology of meditation – place and person factors

We argue that the lack of clear research outcomes from such meditation research is attributable to methodological shortcomings in research design, a consequence of an emphasis on technological considerations and a neglect of critical subjective factors impacting on the meditation experience. For example, reporting of meditation experience may require a more detailed description of personal history than has been the norm in previous research. Place variables, such as the context within which classical meditation was developed and practiced may also effect the meditation experience yet are rarely reported in meditation research (15).

The physical setting for the recording session may further impact on the meditation state attained, affecting its “ecological validity.” Factors affecting the meditation state may include whether the setting is a place usually used for meditation or a laboratory, the intrusiveness and discomfort of the psycho-physiological measures, and the presence of others in the meditation space. Early field studies found even advanced Indian yoga meditators were disturbed by the apparatus and did not attain their usual depth of practice (16). Whether the meditation under study is embedded in its original cultural setting or transposed to a western setting may also influence the results.

## Review of Meditation Research

In order to determine the extent and quality of previous meditation state research, we undertook a review of the meditation state literature incorporating EEG measures and attempted to classify the variables examined. This review was not intended to be an exhaustive review of meditation research, rather it was intended to illustrate the current state and direction of meditation research and highlight the main variables used. Our review began with the report of Cahn and Polich (2), a comprehensive review of meditation research undertaken to that date. This provided 35 EEG studies of meditation states. The U.S. National Library of Medicine service PubMed was then searched for studies using search terms “meditation state” AND “EEG” AND date of publication: “2006–2012.” This returned 37 articles.

Studies were included if they were:

- experimental studies with subjects engaged in a specific meditation practice
- included outcome measures that included EEG recordings of the meditation state.

Excluded from the results were a total of 31 articles based on:

- included in initial list of Cahn and Polich: 2
- trait effects of meditation only: 13
- non-EEG studies: 4
- non-meditation studies: 7
- studies on EEG methodology: 3
- non-experimental studies: 2.

After these exclusion criteria were applied, six articles were added to the present review.

The variables used in the studies were assigned on the basis of reported information into the domains of:

- person – characteristics of the meditator
- practice – nature of the specific meditation practice
- place – variables of the experimental situation
- phenomenology – subjective experiences of the meditator
- physiology – EEG measures of the meditation state.

## Results

Tables 2 and 3 summarizes the domains of “Practice,” “Phenomenology,” and “Psychophysiology” for studies from Table 1 that provided a sufficiently detailed description of the meditation practice to enable categorization as either “FA” or “OM.”

**Table 1:** **Context for meditation studies – place and person**.

Study	Place			Person			
	Original culture	Transposed tradition	Secular adaptation	Novice (<1 year)	STM (1–5 year)	LTM (5–20 year)	Adv (>20 year)
Das and Gastaut (17)	Yoga – India						7

Wenger and Bagchi (16)	Yoga – India						14

Anand (18)	Yoga – India						6

Kasamatsu and Hirai (19)	Zen – Japan				20	12	16

Wallace (20)		TM – USA		15			

Banquet (21)		TM – USA		12			

Pagano and Frumkin (22)		TM – USA			5		

Ghista et al. (23)	Ananda Marga – India					4	

Bennett and Trinder (24)		TM – USA			16		

Hebert and Lehmann (25)		TM – Switzerland			78		

Morse et al. (3)			Mantra (TM), hypnosis, relaxation – USA	12			

Fenwick et al. (26)		TM – USA			2		

Elson et al. (27)		Ananda Marga – USA			11		

Corby et al. (28)		Ananda Marga – USA			20		

Lehrer et al. (29)			CSM, PR – USA	10			

Stigsby et al. (30)		TM – Denmark			14		

Becker and Shapiro (31)		TM, Zen, Yoga – USA			10 Zen,	Yoga, TM	

Dillbeck and Bronson (32)		TM – USA		15			

Farrow and Hebert (33)		TM – USA			28		

Badawi et al. (34)		TM – USA			54		

Zhang et al. (35)	Qigong – China					7	

Gaylord et al. (36)			TM, PR – USA	25 TM, 29PR			

Benson et al. (37)	Tibetan Buddhist – India						2

Pan et al. (38)	Qigong – China				50		

Travis and Wallace (39)		TM – USA			20		

Dunn et al. (40)			Concentration vs. Mindfulness – USA	10			

Kamei (41)		Yoga – Japan				8	

Khare and Nigam (42)	Yoga, TM – India			30			

Arambula et al. (43)	Kundalini Yoga – Japan						1

Litscher et al. (44)	Qigong (Austria)						2

Travis (45)		TM – USA			30		

Lehmann et al. (46)	Tibetan Buddhist (Switzerland)						1

Ott (10)		Yoga, TM – Germany				8 TM, 2 Yoga	

Aftanas and Golocheikine (47), Aftanas and Golocheikine (48, 49)		Sahaja Yoga – Russia		11	16		

Lo et al. (50)	Zen – Taiwan					2	

Lutz et al. (51)	Tibetan Buddhist (USA)					8	

Faber (52)	Zen (Switzerland)					1	

Murata et al. (53)			Zen Susoku (Japan)	22			

Takahashi et al. (54)			Zen Susoku (Japan)	20			

Hebert et al. (55)		TM – USA					15

Chan et al. (56)	TBRT – Hong Kong			19			

Beauregard and Paquette (57)	Christian – Canada					18	

Huang and Lo (58)	Zen – Taiwan				23		

Lagopoulos et al. (59)			Non-directive meditation (Australia)				

Cahn et al. (60)		Vipassana – USA					16

Lehmann et al. (61)	Tibetan Buddhist, Qigong, Sahaja Yoga, Ananda Marga, Zen						

Travis (62)		TM – USA					26

*CSM, clinically standardized meditation; PR, progressive relaxation; SRM, self-regulation method; AT, autogenic training; RR, relaxation response, TBRT, triarchic body-pathway relaxation technique*.

**Table 2:** **Studies using focused attention meditation**.

Study	Practice				Phenomenology		Psychophysiology – EEG bands			
	Practice	Description	Eyes	Focus	Description	Subjective report	θ (4–8 Hz)	α (8–12 Hz)	β (13–30 Hz)	γ (30–50 Hz)
Kasamatsu and Hirai (19) Japan	Zen	Zazen	Open	NR	“Concentration without tension” “special state of consciousness”	Informal	Bursts in advanced	Power increase freq decrease	Not measured?	Not measured?

Ghista et al. (23) India	Ananda Marga	“Intuitional practice”	Closed	Chakra, personal mantra	“Distinct from mental concentration”	No	Power increase	Power increase freq decrease	Not measured	Not measured

Elson et al. (27) USA	Ananda Marga		Closed	Personal mantra	“Mental withdrawal and concentration”	No	Power increase in advanced	Power increase	Not measured	Not measured

Corby et al. (28) USA	Ananda Marga	Tantric yoga	Closed	Personal mantra, with breath	“Intense concentration of attention”	Meditation quality rating	Power increase with proficiency	Some power increase	Not measured	Not measured

Pan et al. (38) China	Qigong	Concentrative	Closed	Attention on breath or body sensation	Thinking regulation	No	Frontal power increase	Power increase	NR	Not measured

Kamei (41) Japan	Yoga	SoHam, preceded by asana, pranayama	Closed	Breath, mantra	NR	No	Not reported	Power increase in most S’s	Not reported	Not reported

Lehmann et al. (46) Switzerland	Diamond Way Buddhist	Buddhist	Half closed	Visualization	Subjectively different meditations	Informal	Not measured	Not measured	Not measured	Right posterior
				Mantra						Left central
				Self						Right anterior

Lo et al. (50) Taiwan	Zen	Inner light	Closed	Zen and third eye chakra	Perception of “inner light”	Signaling of “inner light”	Not reported	Power increase in early meditation	Occurrence on perception of “inner light”	Not reported

Kubota et al. (66) Japan	Zen	Susoku	Open	Counting breath	“Concentrated but relaxed”	Signaling of count	Some frontal found	Occipital – no difference	Not measured	Not measured

Murata et al. (53) Japan	Zen	Susoku	Open, lying down	Counting breath	“Concentrating the mind”	Signaling of count	No difference	Frontal coherence increase	No difference	Not measured

Takahashi et al. (54) Japan	Zen	Susoku	Open	Counting breath	“Concentrating the mind”	Signaling of count	Frontal power increase	Central power increase	No difference	Not measured

Huang and Lo (58) Taiwan	Zen		Closed	Zen chakra	“Samadhi”	Post-session questionnaire		Increase at start	Increased with meditation concentration	Not measured

**Table 3:** **Studies using open monitoring meditation**.

Study	Practice				Phenomenology		Psychophysiology – EEG bands			
	Practice	Description	Eyes	Focus	Description	Subjective report	θ (4–8 Hz)	α (8–12 Hz)	β (13–30 Hz)	γ (30–50 Hz)
Aftanas and Golocheikine (47), Aftanas and Golocheikine (48, 49) Siberia	Sahaja yoga		Closed	Loose, unfixed attention	“Thoughtless awareness and bliss”	Post-session questionnaire	Coherence greater for LTM, bliss, no thought	Lower power	Not measured	Not measured

Lutz et al. (51) USA	Tibetan Buddhist	“loving-kindness”	Not stated	Open	“Pure compassion”	No	Not reported	Not reported	Not reported	Power increased

Chan et al. (56) Hong Kong	Triarchic Body-pathway Relaxation	Mindfulness	Closed	“Attending to thoughts and sensations”	“Deep relaxation and internalized attention”	No	Frontal power increase	More left activation	Not measured	Not measured

Cahn et al. (60)	Vipassana	Scanning of sensations	Closed	Open	“Detached observation”	Post-session questionnaire	Decreased bilateral frontal delta power. No theta effects	Occipital alpha power somewhat related to expertise	Not reported	Increase in parieto-occipital gamma

Responding to the categories proposed by Lutz et al. (11), Travis and Shear (63) proposed a third category of “automatic self-transcending,” marked by the “absence of both (a) focus and (b) individual control or effort” (p2), particularly applicable to “Transcendental Meditation” (TM).

## Results/Discussion

### “Place” variables

“Place” encompasses those variables defining the broad context for the experimental study. Table 1 shows that early studies in the 1960s were conducted in field settings in countries of origin of the meditation tradition studied. The 1970s and 1980s were dominated by studies conducted in USA of Transcendental Meditation, a westernized yoga practice. More recent studies have taken advantage of the increased access in western countries to meditators trained within original eastern meditation traditions. Yet, it is suggested that even our ordinary state of consciousness is in part a product of consensus reality, structured by our cultural context and that the process of “enculturation” during childhood development shapes our experience of consciousness (64). Thus, the cultural history of meditation tradition and practice within a particular society may determine the broad range of experiences available to meditators. This aspect of meditation research has yet to be explored and to our knowledge there have been no comparisons of meditation states attained in different settings.

### “Person” variables

This domain encompasses variables relating to the personal history and the nature of the meditation training of participants. Apart from the extensive work conducted on TM, most studies appear to have selected the meditation practices to study on the basis of availability of subjects. Table 1 shows that early field studies used advanced practitioners, while the western studies of the 1970s–1990s usually had meditators with <5 years practice. More recent studies have again had access to experienced meditators.

As yet there is no accepted way for researchers to determine meditation proficiency. “Years of meditation practice” has been the primary index of meditation expertise used in meditation research, but this represents only a crude measure of proficiency. The stage of life when the practice was performed may also be relevant as brain structures are more malleable in the formative years. In traditional contemplative practice, a number of meditation practices may be utilized (7), thus attempting to specify the total hours spent in a particular meditation practice is not straightforward.

Unlike other activities such as musical performance or video gaming, which lend themselves to more objective measures such as skill ranking or professional and educational attainment (65), meditation is an internal subjective experience that does not lend itself to external rating. A more accurate proficiency measure, although not so easily obtained, is a rating by the meditator’s teacher such as used by Kasamatsu and Hirai (19).

Ideally studies should encompass a range of meditation proficiency – beginners can show the progressive specificity of the effects of meditation, while experienced meditators are more likely to show distinct changes in states of consciousness (13). Further collaboration between researchers and specific meditation traditions is needed to develop classifications of expertise and levels of training required for proficiency within each tradition.

In contrast to traditionally trained meditators, there is some evidence that western meditators may have a more varied journey through their meditation training, often sampling different teachings before settling within one tradition. A qualitative study of western meditators showed that even within a specific meditation tradition (Kashmir Savism) meditators may undertake a switching back and forth between techniques before adopting a technique suitable for that particular meditation session (Abbott, Ph.D. dissertation, University of Houston, 1996).

The intent of the meditator along with the goals and expectations of the participants in research studies may also be important variables. The same meditation practice, performed as part of a long-standing spiritual practice, e.g., Kasamatsu and Hirai (19) or for an undergraduate project, e.g., Dunn et al. (40) may produce very different states. This issue has received little attention in the literature.

### “Practice” variables

The aim of meditation state research has often been to distinguish each meditation category by its cognitive processes, which can be linked to associated neurophysiological activity. For example, based on their EEG signatures, Travis and Shear (63) assigned differing meditation practices to particular categories. Yet, as reported in Tables 2–4, the descriptions of the meditation practices provided by most studies are insufficient to enable clear specification of the cognitive tasks involved. Also most studies failed to record or report EEG activity across all frequency bands, undermining the validity of this approach.

**Table 4:** **Studies using transcendental meditation**.

Study	Practice				Phenomenology		Psychophysiology – EEG bands			
	Practice	Description	Eyes	Focus	Description	Subjective report	θ (4–8 Hz)	α (8–12 Hz)	β (13–30 Hz)	γ (30–50 Hz)
Banquet (21)	TM	Mantra	Closed	Internal	Relaxed attention	Push-button code for five psychological states	Second stage: bursts or trains	First stage: power increase, frequency decrease	Third stage: rhythmic waves correlated with “deep meditation”	Not reported

Hebert and Lehmann (25)	TM	Mantra	Closed	Internal	“No concentration”	Subjective state reported when theta bursts observed	Frontal bursts correlated with “drifting”	Background activity	Occasional	Not reported

Morse et al. (3)	TM, hypnosis, relaxation	TM mantra or “one”	Closed	Internal	“Let mind drift”	States compared	Not reported	All states produced increased power, negative correlation with “depth”	Not reported	Not reported

Travis (45)	TM	Not described	Closed	Internal	“Transcending” or “mental and physical activity”	Post-session report at bell ring 5 min intervals as “transcending” or “other”	Not reported	Higher amplitude and coherence with “transcending”	Not reported	Not reported

Hebert et al. (55)	TM	Not described	Closed	Internal	“Restful alertness”	Not obtained	Not reported	Anterior-posterior phase synchrony	Not reported	Not reported

Travis (62)	TM, TM-Siddhi	TM: mantra, general description of TM-Siddhi	Closed	Internal	Not obtained	Not obtained	Not reported	Stronger sources of alpha1 in TM-Siddhi compared to TM	Not reported	Not reported

An alternative approach suggests that meditation training, regardless of the specific tradition, contains common characteristics and stages of development and that irrespective of the tradition, meditative training involves a similar scheme of development that can be related to distinct EEG signatures (13). In contrast, Lutz et al. (7), while not discounting the possibility that practices from different traditions can have similar effects, contend that it is best to avoid this assumption. Instead they recommend that each meditation tradition’s discourse needs to be examined and interpreted to derive descriptions of meditation states that are measureable and repeatable and therefore useful for research. Based on the reviewed studies, it certainly appears that any attempt to link specific meditation practices to EEG signatures is premature and that further progress in this endeavor will require the inclusion of data from the domain of phenomenology.

### “Phenomenology” variables

Tables 2–4 show the few studies that include subjective reports of the meditation states attained, with post-session questionnaires or rating scales being the most used method, e.g., (28, 47). Some studies, e.g., (21) and (45) have attempted in-session reporting of subjective states, but as Lo et al. (50) noted, the intrusiveness of these methods renders them impractical during deep meditation.

A potential strategy to improve the accuracy and validity of subjective reports of inner experience is the research approach advocated by Lutz and Thompson (67) in which first-person data from trained subjects is used to guide third-person neurophysiological measurements. Although this approach has not been widely adopted, advanced meditators are seen as providing a more refined first-person description of their experiences (7).

There have been a number of attempts to develop methods for mapping the phenomenology of altered states of consciousness, applicable to meditation practice. Tart (68) argued that the term “altered state of consciousness” has come to be used too loosely. He proposed it to be replaced by a new term; “discrete altered state of consciousness” (d-ASC), described as “unique, dynamic patterns or configurations of psychological structures, active systems of psychological subsystems” (p5). Meditation-induced states of consciousness form a subset of these states (69) explain that Tart’s notion of “psychological structures” includes those structures, which regulate the basic parameters of consciousness. These structures are revealed by a recognizable isomorphism (not merely a correlation) between phenomenology and physiology. Thus, a d-ASC in meditation would be expressed in a discrete state of brain networks, observable as a change in the dominant network of functional connectivity between brain regions, from a defined baseline state.

In a review of a wide range of altered states of consciousness Vaitl et al. (70), used a four dimensional matrix, assessed predominantly on the self-report of the subjects. The dimensions were: “activation” (high to low arousal), “awareness span” (from narrow to broad), “self-awareness” (from heightened to diminished), and “sensory dynamics” (from reduced to heightened sensation). Ott (10) explored the subjective dimensions of “meditation depth” in a sample of yoga, Buddhist, and TM meditators. A factor analysis of 300 questionnaires revealed three dimensions: “mystical experience” – bliss, contact with a higher force, “nirvana” – absence of thought, total absorption, and “mental and bodily relaxation” – reduction of tension.

The dimensions outlined in these two studies can form the foundation for a more detailed analysis of the phenomenological states attained during meditation, expanding the scope beyond the commonly used dimension of attention focus. Meditation practices can be mapped onto these dimensions and subjective reports from meditators used to validate the profile of each practice. Fell et al. (13) describe this process as defining each meditation state of consciousness as a unique area in state space. Exploring neurophysiological signatures of these states can help to answer the question concerning the uniqueness of meditation states of consciousness.

### “Psychophysiology” variables

Measurement and analysis methods used by meditation state research have changed significantly over the decades, impacting on the interpretation of the brain states being measured. The introduction of neuroimagery techniques, e.g., positron emission tomography (PET), (71) and functional magnetic resonance imagery (fMRI), (72) has greatly extended the ability to document topographical brain activity and neuronal metabolism activity in various meditation states. However, essentially the same methodological considerations apply as with EEG outcome measures.

### Toward a standardized reporting format for meditation state research

In order to address the deficiencies in previous research described above, we propose a comprehensive methodological framework for research into meditation states of consciousness to address variables from a broader range of domains.

The first two proposed domains provide the context for the meditation session under study. They are:

- place – the relationship of the study to the cultural origin of the meditation practice and the physical nature of the experimental setting. Variables include; the GPS coordinates of the study location; a description of the setting (laboratory, home, temple, or other facility); the familiarity of the meditator with the setting; the time and date of the data collection, the ambient temperature, barometric pressure, humidity and lighting; the décor including the presence of images, icons, statues, mandalas, yantras, candles, incense, etc.; the proximity to electrical appliances and external electromagnetic fields, the presence of any shielding (Faraday cage), and measures of electromagnetic field strength.
- person – variables relating to the personal history, meditation training, meditation practice, expectations, and motivation of the meditator. This includes; demographic details such as age, gender, ethnicity, socio-economic status, level of education and handedness; health status including the presence or absence of any acute or chronic diseases; anthropometric measures such as height, weight, BMI, head, and waist circumference; current and previous history of use of, or abstinence from, substances such as caffeine, tobacco, alcohol, animal products, and pharmaceutical medicines; details of meditation training including age at commencement of training, type of training, regularity, and duration of practice; the briefing provided prior to the experimental session along with the method of recruitment and personal motivation and expectations of the practitioner. “Person” variables also include measures of personality and temperament such as the Cloninger Temperament and Character Inventory, which has been shown to correlate with meditation performance (53, 54).

A further three domains provide the experimental framework within the particular cultural and personal context.

Practice – the actual meditation practice, described in sufficient detail to allow replication. It is suggested that a minimal description of meditation practice should include the specific lineage and tradition that the practice is based on along with any traditional descriptions: posture, eye attitude (open/closed), and how attention is directed.
Phenomenology – the phenomenal state of consciousness produced by the particular meditation practice, as predicted by meditation teachings and the actual experience in the session, validated by feedback from the participants. Further development of methods for mapping phenomenological space, building on the work of Vaitl et al. (70) would enable a standardized format to be adopted. Other measures, including “absorption,” measured by the Tellegen Absorption Scale also warrant further investigation for usefulness in meditation research (73).
Psychophysiology – adequate documentation of equipment used, methods of collection, analysis, and interpretation of psycho-physiological measures. These measures may include: cardiovascular performance, e.g., heart rate, blood pressure, heart rate variability (HRV) (74); brain activity measures, e.g., EEG frequency and low resolution topographical analysis (LORETA) (46, 75); and various neuroimaging measures, e.g., MRI to measure cortical thickness (76); Single Photo Emission Computerized Tomography (SPECT) to measure cerebral blood flow (77) and other techniques such as the combined use of EEG (for temporal definition) and fMRI (for spatial definition) (78).

We suggest that the value of this proposed framework would be an improved ability to conduct reproducible research that can accurately position specific meditation states within the total matrix of states of consciousness. By placing the specific relationship of practice-phenomenal state-psycho-physiological measure within the broader context of “place” and “person,” the research questions posed in the introduction can be more confidently addressed: do meditation states have specific psycho-physiological characteristics and do meditation states induce specific states of consciousness?

While this approach may lead to more reproducible research and therefore more consistent results, it does not provide specific direction for the formulation of research hypotheses. As the aim of meditation is the experience of mystical higher states of consciousness beyond the thinking mind, it may be that some aspects of meditation are not amenable to rational scientific inquiry and can only be explored through direct personal experience. Nevertheless, it is hoped that the implementation of a consistent approach to reporting research data will advance meditation research and thereby assist in penetrating deeper into the mysteries of human consciousness.

## Conclusion

Research to date into meditation states has been inconclusive and is hampered by a number of methodological limitations, primarily the narrow range of variables included in research designs and the lack of inclusion of phenomenological data. Within the context provided by the “place,” “person,” and “practice,” we argue that research will be advanced by a comprehensive program of mapping of phenomenological states to meditation practices and then to psycho-physiological variables. Given this foundation, the questions of uniqueness of meditation states and the specificity of meditation effects may begin to be addressed.

## Conflict of Interest Statement

The authors declare that the research was conducted in the absence of any commercial or financial relationships that could be construed as a potential conflict of interest.

## References

[1] Cohen MM Penman S Pirotta M Da Costa C The integration of complementary therapies in Australian general practice: results of a national survey. J Altern Complement Med (2005) 11:995–1004 10.1089/acm.2005.11.995 16398590

[2] Cahn BR Polich J Meditation states and traits: EEG, ERP, and neuroimaging studies. Psychol Bull (2006) 132:180–211 10.1037/0033-2909.132.2.180 16536641

[3] Morse DR Martin JS Furst ML Dubin LL A physiological and subjective evaluation of meditation, hypnosis, and relaxation. Psychosom Med (1977) 39:304–24 10.1097/00006842-197709000-00004 333497

[4] Shapiro DH Jr Overview: clinical and physiological comparison of meditation with other self-control strategies. Am J Psychiatry (1982) 139:267–74 7036760 10.1176/ajp.139.3.267

[5] Rubia K The neurobiology of meditation and its clinical effectiveness in psychiatric disorders. Biol Psychol (2009) 82:1–11 10.1016/j.biopsycho.2009.04.003 19393712

[6] West MA Meditation and the EEG. Psychol Med (1980) 10:369–75 10.1017/S0033291700044147 6992184

[7] Lutz A Dunne JD Davidson RJ Meditation and the neuroscience of consciousness. In: Zelazo P, editor. Cambridge Handbook of Consciousness. Cambridge: Cambridge University Press (2007). p. 499–554

[8] Ospina MB Bond K Karkhaneh M Tjosvold L Vandermeer B Liang Y Meditation practices for health: state of the research. Evid Rep Technol Assess (Full Rep) (2007) 155:1–263 17764203 PMC4780968

[9] Bond K Ospina M Hooton N Shannahoff-Khalsa D Dusek J Carlson L Defining a complex intervention: the development of demarcation criteria for “meditation”. Psychol Religion Spiritual (2009) 1:129–37 10.1037/a0015736

[10] Ott U The EEG and depth of meditation. J Meditation Med Res (2001) 1:55–68

[11] Lutz A Slagter HA Dunne JD Davidson RJ Attention regulation and monitoring in meditation. Trends Cogn Sci (2008) 12:163–9 10.1016/j.tics.2008.01.005 18329323 PMC2693206

[12] Woolfolk RL Psychophysiological correlates of meditation. Arch Gen Psychiatry (1975) 32(10):1326–33 10.1001/archpsyc.1975.01760280124011 1180661

[13] Fell J Axmacher N Haupt S From alpha to gamma: electrophysiological correlates of meditation-related states of consciousness. Med Hypotheses (2010) 75:218–24 10.1016/j.mehy.2010.02.025 20227193

[14] Walsh R An evolutionary model of meditation research. In: Shapiro D, editor. Meditation: Classic and Contemporary Perspectives. New York: Aldine (1984).

[15] Deikman AJ The state of the art of meditation. In: Shapiro DAW, editor. Meditation: Classic and Contemporary Perspectives. New York: Aldine (1984). p. 679–80

[16] Wenger M Bagchi B Studies of autonomic functions in practitioners of yoga in India. Behav Sci (1961) 6:312–23 10.1002/bs.3830060407 14006122

[17] Das N Gastaut H Variations de l’activite electrique du cerveau, du couer et des muscles an cours de la meditation et de l’extrase yogique. Electroceph. Clin. Neurophysiol. Supp (1957) 6:211–9

[18] Anand B Some aspects of electroencephalographic studies in yogis. Electroencephalogr Clin Neurophysiol (1961) 13:452–6 10.1016/0013-4694(61)90015-3

[19] Kasamatsu A Hirai T An electroencephalographic study on the Zen meditation (Zazen). Folia Psychiatr Neurol Jpn (1966) 20:315–36 6013341 10.1111/j.1440-1819.1966.tb02646.x

[20] Wallace RK Physiological effects of transcendental meditation. Science (1970) 167:1751–4 10.1126/science.167.3926.1751 5416544

[21] Banquet JP Spectral analysis of the EEG in meditation. Electroencephalogr Clin Neurophysiol (1973) 35:143–51 10.1016/0013-4694(73)90170-3 4124606

[22] Pagano RR Frumkin LR The effect of transcendental meditation on right hemispheric functioning. Biofeedback Self Regul (1977) 2:407–15 10.1007/BF00998625 348241

[23] Ghista DN Nandagopal D Ramamurthi B Das A Mukherji A Srinivasan TM Physiological characterisation of the ‘meditative state’ during intuitional practice (the Ananda Marga system of meditation) and its therapeutic value. Med Biol Eng (1976) 14(2):209–13 10.1007/BF02478750 781406

[24] Bennett JE Trinder J Hemispheric laterality and cognitive style associated with transcendental meditation. Psychophysiology (1977) 14:293–6 10.1111/j.1469-8986.1977.tb01178.x 323902

[25] Hebert R Lehmann D Theta bursts: an EEG pattern in normal subjects practising the transcendental meditation technique. Electroencephalogr Clin Neurophysiol (1977) 42:397–405 10.1016/0013-4694(77)90176-6 65275

[26] Fenwick P Donaldson S Gillis L Bushman J Fenton G Perry I Metabolic and EEG changes during transcendental meditation: an explanation. Biol Psychol (1977) 5:101–18 10.1016/0301-0511(77)90007-2 328063

[27] Elson BD Hauri P Cunis D Physiological changes in yoga meditation. Psychophysiology (1977) 14:52–7 10.1111/j.1469-8986.1977.tb01155.x 319471

[28] Corby JC Roth WT Zarcone VP Jr. Kopell BS Psychophysiological correlates of the practice of Tantric yoga meditation. Arch Gen Psychiatry (1978) 35:571–7 10.1001/archpsyc.1978.01770290053005 365124

[29] Lehrer PM Woolfolk RL Rooney AJ McCann B Carrington P Progressive relaxation and meditation. A study of psychophysiological and therapeutic differences between two techniques. Behav Res Ther (1983) 21:651–62 10.1016/0005-7967(83)90083-9 6362649

[30] Stigsby B Rodenberg JC Moth HB Electroencephalographic findings during mantra meditation (transcendental meditation). A controlled, quantitative study of experienced meditators. Electroencephalogr Clin Neurophysiol (1981) 51:434–42 10.1016/0013-4694(81)90107-3 6164542

[31] Becker DE Shapiro D Physiological responses to clicks during zen, yoga, and TM meditation. Psychophysiology (1981) 18:694–9 10.1111/j.1469-8986.1981.tb01846.x 7031742

[32] Dillbeck MC Bronson EC Short-term longitudinal effects of the transcendental meditation technique on EEG power and coherence. Int J Neurosci (1981) 14:147–51 10.3109/00207458108985827 7030999

[33] Farrow J Hebert J Breath suspension during the transcendental meditation technique. Psychosomatic Medicine (1982) 44:133–53 7045911 10.1097/00006842-198205000-00001

[34] Badawi K Wallace RK Orme-Johnson D Rouzere A Electrophysiologic characteristics of respiratory suspension periods occurring during the practice of TM program. Psychosom Med (1984) 46:267 10.1097/00006842-198405000-00008 6377350

[35] Zhang J-Z Jing Z He Q-N EEG findings during special psychical state (Qigong state) by means of compressed spectral array and topograhical mapping. Comput Biol Med (1988) 18:455–63 10.1016/0010-4825(88)90063-7 3060312

[36] Gaylord C Orme-Johnson D Travis F The effects of the transcendental mediation technique and progressive muscle relaxation on EEG coherence, stress reactivity, and mental health in black adults. Int J Neurosci (1989) 46:77–86 10.3109/00207458908991618 2670798

[37] Benson H Malhotra MS Goldman RF Jacobs GD Hopkins PJ Three case reports of the metabolic and electroencephalographic changes during advanced Buddhist meditation techniques. Behav Med (1990) 16:90–5 10.1080/08964289.1990.9934596 2194593

[38] Pan W Zhang L Xia Y The difference in EEG theta waves between concentrative and non-concentrative qigong states – a power spectrum and topographic mapping study. J Tradit Chin Med (1994) 14:212–8 7799657

[39] Travis F Wallace RK Autonomic and EEG patterns during eyes-closed rest and transcendental meditation (TM) practice: the basis for a neural model of TM practice. Conscious Cogn (1999) 8:302–18 10.1006/ccog.1999.0403 10487785

[40] Dunn BR Hartigan JA Mikulas WL Concentration and mindfulness meditations: unique forms of consciousness? Appl Psychophysiol Biofeedback (1999) 24:147–65 10.1023/A:1023498629385 10652635

[41] Kamei T Decrease in serum cortisol during yoga exercise is correlated with alpha wave activity. Percept Mot Skills (2000) 90:1027–32 10.2466/pms.2000.90.3.1027 10883793

[42] Khare KC Nigam SK A study of electroencephalogram in meditators. Indian J Physiol Pharmacol (2000) 44:173–8 10846631

[43] Arambula P Peper E Kawakami M Gibney KH The physiological correlates of Kundalini yoga meditation: a study of a yoga master. Appl Psychophysiol Biofeedback (2001) 26:147–53 10.1023/A:1011343307783 11480165

[44] Litscher G Wenzel G Niederwieser G Schwarz G Effects of QiGong on brain function. Neurol Res (2001) 23:501–5 10.1179/016164101101198749 11474806

[45] Travis F Autonomic and EEG patterns distinguish transcending from other experiences during Transcendental Meditation practice. Int J Psychophysiol (2001) 42:1–9 10.1016/S0167-8760(01)00143-X 11451476

[46] Lehmann D Faber PL Achermann P Jeanmonod D Gianotti LRR Pizzagalli D Brain sources of EEG gamma frequency during volitionally meditation-induced, altered states of consciousness, and experience of the self. Psychiatry Res Neuroimaging (2001) 108:111–21 10.1016/S0925-4927(01)00116-0 11738545

[47] Aftanas LI Golocheikine SA Human anterior and frontal midline theta and lower alpha reflect emotionally positive state and internalized attention: high-resolution EEG investigation of meditation. Neurosci Lett (2001) 310:57–60 10.1016/S0304-3940(01)02094-8 11524157

[48] Aftanas LI Golocheikine SA Non-linear complexity of the human EEG during meditation. Neurosci Lett (2002) 330(2):143–6 10.1016/S0304-3940(02)00745-0 12231432

[49] Aftanas LI Golocheikine SA Changes in cortical activity during altered states of consciousness: study of meditation by high resolution EEG. Hum Physiol (2003) 29(2):143–51 10.1023/A:1022986308931 12751217

[50] Lo PC Huang ML Chang KM EEG alpha blocking correlated with perception of inner light during Zen meditation. Am J Chin Med (2003) 31:629–42 10.1142/S0192415X03001272 14587885

[51] Lutz A Greischar LL Rawlings NB Ricard M Davidson RJ Long-term meditators self-induce high-amplitude gamma synchrony during mental practice. Proc Natl Acad Sci U S A (2004) 101:16369–73 10.1073/pnas.0407401101 15534199 PMC526201

[52] Faber PL Scalp and Intracerebral (LORETA) Theta and Gamma EEG Coherence in Meditation. Winterhur: European Chapter of Int. Soc Neuronal Regulation (2004).

[53] Murata T Takahashi T Hamada T Omori M Kosaka H Yoshida H Individual trait anxiety levels characterizing the properties of zen meditation. Neuropsychobiology (2004) 50:189–94 10.1159/000079113 15292676

[54] Takahashi T Murata T Hamada T Omori M Kosaka H Kikuchi M Changes in EEG and autonomic nervous activity during meditation and their association with personality traits. Int J Psychophysiol (2005) 55:199–207 10.1016/j.ijpsycho.2004.07.004 15649551

[55] Hebert R Lehmann D Tan G Travis F Arenander A Enhanced EEG alpha time-domain phase synchrony during transcendental meditation: implications for cortical integration theory. Signal Processing (2005) 85:2213–32 10.1016/j.sigpro.2005.07.009

[56] Chan AS Han YM Cheung MC Electroencephalographic (EEG) measurements of mindfulness-based Triarchic body-pathway relaxation technique: a pilot study. Appl Psychophysiol Biofeedback (2008) 33:39–47 10.1007/s10484-008-9050-5 18214668

[57] Beauregard M Paquette V EEG activity in Carmelite nuns during a mystical experience. Neurosci Lett (2008) 444:1–4 10.1016/j.neulet.2008.08.028 18721862

[58] Huang H-Y Lo P-C EEG dynamics of experienced Zen meditation practitioners probed by complexity index and spectral measure. J Med Eng Technol (2009) 33:314–21 10.1080/03091900802602677 19384707

[59] Lagopoulos J Xu J Rasmussen I Vik A Malhi GS Eliassen CF Increased theta and alpha EEG activity during nondirective meditation. J Altern Complement Med (2009) 15:1187–92 10.1089/acm.2009.0113 19922249

[60] Cahn BR Delorme A Polich J Occipital gamma activation during Vipassana meditation. Cogn Process (2010) 11:39–56 10.1007/s10339-009-0352-1 20013298 PMC2812711

[61] Lehmann D Faber PL Tei S Pascual-Marqui RD Milz P Kochi K Reduced functional connectivity between cortical sources in five meditation traditions detected with lagged coherence using EEG tomography. Neuroimage (2012) 60:1574–86 10.1016/j.neuroimage.2012.01.042 22266174

[62] Travis F Comparison of coherence, amplitude, and eLORETA patterns during Transcendental Meditation and TM-Sidhi practice. Int J Psychophysiol (2011) 81:198–202 10.1016/j.ijpsycho.2011.06.011 21726586

[63] Travis F Shear J Focused attention, open monitoring and automatic transcending: categories to organise meditations from Vedic, Buddhist and Chinese traditions. Conscious Cogn (2010) 19(4):1110–8 10.1016/j.concog.2010.01.007 20167507

[64] Tart C States of Consciousness. New York: E.P. Dutton (1975).

[65] Latham A Patston LL Tippett LJ Just how expert are “expert” video-game players? Assessing the experience and expertise of video-game players across “action” video-game genres. Front Psychol (2013) 4:941 10.3389/fpsyg.2013.00941 24379796 PMC3863720

[66] Kubota Y Sato W Toichi M Murai T Okada T Hayashi A Frontal midline theta rhythm is correlated with cardiac autonomic activities during the performance of an attention demanding meditation procedure. Brain Res Cogn Brain Res (2001) 11(2):281–7 11275489 10.1016/s0926-6410(00)00086-0

[67] Lutz A Thompson E Neurophenomenology: integrating subjective experience and brain dynamics in the neuroscience of consciousness. J Conscious Stud (2003) 10:31–52

[68] Tart CT States of Consciousness. Lincoln: iUniverse.com Inc (2000). [Original work published 1983].

[69] Jamieson G Hasegawa H New paradigms of hypnosis research. In: Jamieson G, editor. Hypnosis and Conscious States: The Cognitive Neuroscience Perspective. Oxford: Oxford University Press (2007). p. 133–44

[70] Vaitl D Gruzelier J Jamieson G Lehmann D Ott U Sammer G Psychobiology of altered states of consciousness. Psych Bull (2005) 131:98–127 10.1037/0033-2909.131.1.98 15631555

[71] Lou HC Kjaer TW Friberg L Wildschiodtz G Holm S Nowak M A 15O-H2O PET study of meditation and the resting state of normal consciousness. Hum Brain Mapp (1999) 7:98–105 10.1002/(SICI)1097-0193(1999)7:2<98::AID-HBM3>3.3.CO;2-D 9950067 PMC6873339

[72] Hölzel BK Ott U Hempel H Hackl A Wolf K Stark R Differential engagement of anterior cingulate and adjacent medial frontal cortex in adept meditators and non-meditators. Neurosci Lett (2007) 421:16–21 10.1016/j.neulet.2007.04.074 17548160

[73] Jamieson G The Modified Tellegen Absorption Scale: a clearer window on the structure and meaning of absorption. Aust J Clin Exp Hypn (2005) 33:119–39

[74] Krygier JR Heathers JA Shahrestani S Abbott M Gross JJ Kemp AH Mindfulness meditation, well-being and heart rate variability: a preliminary investigation into the impact of intensive Vipassana meditation. Int J Psychophysiol (2013) 89:305–13 10.1016/j.ijpsycho.2013.06.017 23797150

[75] Travis F Haaga D Hagelin J A self-referential default brain state: patterns of coherence, power and eLORETA sources during eyes-closed rest and Transcendental Meditation. Cogn Process (2010) 11:21–30 10.1007/s10339-009-0343-2 19862565

[76] Grant JA Duerden EG Courtemanche J Cherkasova M Duncan GH Rainville P Cortical thickness, mental absorption and meditative practice: possible implications for disorders of attention. Biol Psychol (2013) 92:275–81 10.1016/j.biopsycho.2012.09.007 23046904

[77] Newberg A Alavi A Baime M Pourdehnad M Santanna J d’Aquili E The measurement of regional cerebral blood flow during the complex cognitive task of meditation: a preliminary SPECT study. Psychiatry Res (2001) 106:113–22 10.1016/S0925-4927(01)00074-9 11306250

[78] Beauregard M Courtemanche J Paquette V Brain activity in near-death experiencers during a meditative state. Resuscitation (2009) 80:1006–10 10.1016/j.resuscitation.2009.05.006 19573975

